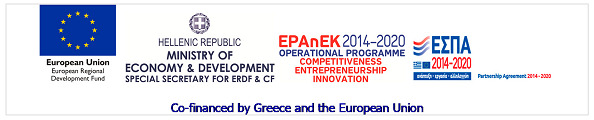# Quercetin and Egg Metallome

**DOI:** 10.3390/antiox10010080

**Published:** 2021-01-09

**Authors:** Evangelos Zoidis, Athanasios C. Pappas, Michael Goliomytis, Panagiotis E. Simitzis, Kyriaki Sotirakoglou, Savvina Tavrizelou, George Danezis, Constantinos A. Georgiou

**Affiliations:** 1 Laboratory of Nutritional Physiology and Feeding, Department of Animal Science, Agricultural University of Athens, 11855 Athens, Greece; apappas@aua.gr (A.C.P.); stud217094@aua.gr (S.T.); 2 Laboratory of Animal Breeding and Husbandry, Department of Animal Science, Agricultural University of Athens, 11855 Athens, Greece; mgolio@aua.gr (M.G.); pansimitzis@aua.gr (P.E.S.); 3 Laboratory of Mathematics and Statistics, Department of Natural Resources Management and Agricultural Engineering, Agricultural University of Athens, 11855 Athens, Greece; sotirakoglou@aua.gr; 4 Chemistry Laboratory, Department of Food Science and Human Nutrition, Agricultural University of Athens, 11855 Athens, Greece; gdanezis@aua.gr (G.D.); cag@aua.gr (C.A.G.); 5 FoodOmics.GR Research Infrastructure, Agricultural University of Athens, 11855 Athens, Greece

**Keywords:** egg, flavonoids, inductively coupled plasma mass spectrometry (ICP-MS), metallome, quercetin

## Abstract

The objective of the present study was to investigate the effect of the natural flavonoid quercetin dietary supplementation on the alteration of egg metallome by applying the basic principles of elemental metabolomics. One hundred and ninety-two laying hens were allocated into 4 treatment groups: the control (C) group that was fed with a commercial basal diet and the other experimental groups that were offered the same diet further supplemented with quercetin at 200, 400 and 800 mg per kg of feed (Q2, Q4 and Q8 group, respectively) for 28 days. The diets contained the same vitamin and mineral premix, thus all birds received the same amount of elements since no differences on feed intake existed. The egg elemental profile consisted of As, Ca, Cd, Co, Cr, Cu, Fe, Mg, Mn, Mo, Ni, Pb, Sb, Se, Sr, V, Zn and was determined using inductively coupled plasma mass spectrometry (ICP-MS). Quercetin supplementation altered the elemental profile. Most notably, quercetin altered the element concentrations predominantly in egg shell and albumen. It increased the concentration of Sb while reduced that of Cr and Se in both egg shell and albumen. Moreover, it increased As, Cd in albumen and V in yolk, while compared to the control, reduced As, Cd, Cr, Cu and V and also raised Ca, Fe, Mg and Ni in egg shell. The presence of quercetin led to differentiation of the deposition of certain trace minerals in egg compartments compared to that of hens fed a basal diet, possibly indicating that tailor made eggs for specific nutritional and health requirements could be created in the future.

## 1. Introduction

Consumers are seeking for safe foods that also possess high nutritional value. Much attention has been paid to develop animal products with physiological functions that promote human health. Products of animal origin enriched with bioactive compounds seem to have improved quality characteristics [1,2] Furthermore, fortified animal products may protect consumers against oxidation and spoilage originating from bacteria [1,2]. Several functional foods have been created including but not limited to eggs enriched with ω-3 fatty acids, vitamin E, selenium and vitamin D.

Polyphenols present potential beneficial effects in humans and animals. Flavonoids, a class of polyphenols, are antioxidants of natural origin and they possess anti-inflammatory, antioxidant, hepatoprotective, antibacterial and anticarcinogenic properties in several animal models [3] Most notably, poultry production and health have been affected positively by quercetin [3,4]. Particularly, quercetin strengthens immune system via lymphocyte and macrophage stimulation and antibody production [4,5]. It has been shown to possess anti-inflammatory activity in animals [6,7]) and immunoregulatory effects through enhancing IgY antibody production, weight of lymphoid organs (spleen, thymus and bursa) and natural killer cell activity thereby meliorating productive performance [8]. Flavonoids supplementation in hens’ diet can also improve nutritional, sensorial and microbiological properties of eggs [1].

Molecular formula of quercetin is C_15_H_10_O_7_. Quercetin belongs to flavonols, subclass of flavonoids and it is widely found in different varieties of vegetables and fruits such as onions, apples and their by-products [9,10]. Quercetin properties on human health are recognized by EFSA [11]. Thus, quercetin contributes to cardiovascular, nervous system, brain, liver and kidney health. Furthermore, it supports healthy ageing and may help strengthen body’s defenses [9,11]. Quercetin has antioxidant activities which are important in the control of autoimmune diseases such as pulmonary hypertension syndrome (PHS) in broilers [12]. It plays an important role in improving bone mineral density and volume, suggesting that it influences the micro-architecture of bone tissue [13]. Moreover, quercetin presents further biological actions such as antibacterial, hepatoprotective, growth promoter, antiallergic, antiviral (influenza A virus and rhinovirus), and antithrombotic activities in various animal models [4,14].

Flavonoids could affect metal homeostasis and cellular oxidative status due to dietary flavonoids interactions with trace minerals and flavonoids effect on metallothionein level [15]. Flavonoids display antioxidant characteristics via chelation with transition metals. Metal-flavonoids chelates are more effective free radical scavengers than the parent flavonoids and protect from oxidative stress [7]. Complexes of flavonoids play an important role in limiting metal bioavailability and suppressing metal toxicity [7].

Quercetin occurs as a glycoside (with linked sugars) or as an aglycone (without linked sugars) [9] and, as mentioned above, binds heavy metals and its chelating characteristics could result in lower metal toxicity. Thus, supplementation with quercetin could be regarded as a feasible approach for heavy metal poisoning [4]. Quercetin-metal complexes also present higher anticancer and antibacterial activities than that of free quercetin [16]. In addition, quercetin is chelated with iron, a crucial trace element for many essential biochemical processes. At the same time, quercetin has various roles in absorption of iron, hepcidin (a key regulator of iron metabolism and mediator of anemia of inflammation) regulation and cellular iron uptake and release. Quercetin’s iron chelation has direct scavenging action against ROS (reactive oxygen species) and lessens iron overload induced by various pathologies [16]. Furthermore, quercetin’s complex with Zn interacts with DNA and exerts antioxidative and anti-tumour activities [17]. As it has been shown in diabetic rats, iron-quercetin complex could reverse oxidative stress and iron deficiency mostly caused by the diabetes but concurrently it induces an imbalance in redistribution of other essential metals [18].

Therefore, it would be interesting to investigate possible alteration of the elemental fingerprint due to quercetin supplementation in various doses. Not only nutritive elements but also toxic elements where studied because as previously mentioned quercetin has presented interactions with many metals. This study is part of a project [19] where quercetin was supplemented to laying hens. Possible alterations in the elemental profile could further enhance the nutritional value (nutritive elements) and safety aspects (toxic elements) of these eggs.

## 2. Materials and Methods

### 2.1. Animals and Experimental Design

Previously, detailed description of management of birds used in the study was provided [19]. Briefly, a total of 192 Lohmann Brown-Classic laying hens (70 weeks old), from the flock kept in the facilities of the experimental station of Agricultural University of Athens, were randomly allocated into 4 treatment groups. Each treatment group consisted of 6 replicate enriched cages with 8 hens each. A commercial basal diet was provided to one group namely control (C) whereas, the same diet with quercetin (MP Biomedicals, LLC, Illkirch, France, 97%) added at 200, 400 and 800 mg per kg of feed was provided to other three groups Q2, Q4 and Q8 group, respectively. The duration of the study was 28 days. All diets were in mash form in order to uniformly mix quercetin in the basal diet. The composition of the basal diet is presented in [19]. Birds of all treatments fed the same diet with the exception of added level of quercetin. The same amount of elements was provided to all birds since the diets contained the same vitamin and mineral premix and no differences on feed intake were noted [19]. Throughout the experimental period, the provision of water was ad libitum. The light regimen was 16 h of continuous light a day. At the end of the trial, after 28 days, a number of eggs, 7 per treatment group, was collected (28 eggs total). Eggs were analyzed for trace and macro-element content. The present study followed the guidelines of the directive 2010/63/EU of the European Parliament and of the Council on the protection of animals used for scientific purposes. The Research Ethics Committee of the Faculty of Animal Science of the Agricultural University of Athens approved the study (code number 38/07022017).

### 2.2. Determination of Egg Trace and Macro Elements

Fifteen elements were determined, namely As, Ca, Cd, Co, Cr, Cu, Fe, Mg, Mn, Ni, Sb, Se, Sr, V and Zn. Various elements were included with several roles, for example Ca as major structural component, Ca and Mg as responsible for activation or signalling, Co, Cr, Cu, Fe, Mn, Ni, Se, V and Zn as components of enzymes or hormones, toxic elements (As and Cd) and other that their functionality is not clearly defined (Sb and Sr). As previously described [20,21], elemental profile was determined through inductively coupled plasma mass spectrometry, ICP-MS (Perkin Elmer, Elan 9000, SCIEX, Toronto, ON, Canada).

Chemicals used were nitric acid (Suprapur^®^, 65% *w/v*, Merck, Darmstadt, Germany) and ICP-MS certified multi-element standards (Inorganic Ventures, NJ, USA). Ultrapure water with a resistance of 18.2 MΩ cm^−1^ obtained from a MilliQ plus system (Millipore, Saint Quentin Yvelines, France) was used in all procedures.

Sample digestion was performed with a microwave-assisted digestion system (CEM, Mars X-Press, Matthews, NC, USA). One grammar (1 g wet weight) of albumen or yolk and 0.1 g of shell were weighted in an analytical balance in a polypropylene tube. Then, 10 mL of HNO_3_ were added to pre-digest samples for 30 min. The resulting sample suspension was transferred quantitatively in the microwave digestion PTFE (polytetrafluoroethylene) vessel. The samples were heated in the microwave accelerated digestion system according the following program: the power was ramped during 20 min from 100 W to 1200 W and held for 15 min. The temperature reached a maximum of 200 °C and followed by a cool-down cycle for 15 min. PTFE vessels were sealed throughout the aforementioned cycle to avoid volatilization losses. Although all samples were completely brought to solution, to disregard any small particle passing optical inspection entering the ICP-MS, solutions were filtered with polyester disposable syringe filters 0.20 μm/15 mm (Chromafil, Macherey-Nagel, Düren, Germany). Before injection in the ICP-MS, sample solutions were diluted, as required, with ultrapure water.

Operating conditions of the ICP-MS were as follows: nebulizer gas flow of 0.91 L min^−1^, ICP RF (radio-frequency) power of 1050 W, lens voltage of 8.5 V and pulse stage voltage of 950 V. Calibration curves were prepared from standard solutions of high purity standards.

### 2.3. Statistical Analysis

Data were analyzed using the Statgraphics Centurion statistical package (version 16.1) and are presented as means ± pooled standard errors. Treatment effects on element concentrations were explored using one-way analysis of variance (ANOVA) followed by Duncan’s multiple range test. Kolmogorov-Smirnov test revealed that all variables followed the normal distribution. Principal component analysis (PCA) was also applied in order to reduce the dimensionality of the data and investigate the relationships between the trace and macro elements. Moreover, data of albumen, yolk and shell were subjected to discriminant analysis to investigate samples distinguishment to the four dietary treatments and establishment of those elements capable to distinguish and classify the samples. Selection of discriminant variables was done using Wilk’s lambda (λ) criterion. For all tests, the significance level was set at 5%.

## 3. Results

### 3.1. Concentration of Egg Elements

Concentrations of selected trace- and macro-elements in egg albumen are illustrated in Table 1. Addition of 200 mg quercetin per kg of feed, increased the concentration of Cd (34%) compared to control treatment. Higher levels of quercetin (400 mg/kg) significantly increased the concentration of As (18%) and decreased that of Cr (9%) and Se (23%) compared to that of control. Most notably, feed supplementation with quercetin at a concentration of 800 mg/kg, increased, compared to control, the concentration of As (24%), Cd (32%) and Sb (140-fold) and reduced the concentrations of Cr (15%) and Se (34%) (Table 1).

Egg yolk elements’ concentrations are shown in Table 2. Addition of quercetin significantly increased V levels (ca. 7.5–9%), compared to control. Moreover, quercetin added to the diet at 400 and 800 mg/kg markedly increased, compared to control, the concentration of Sb 7.5-fold and 8-fold, respectively (Table 2).

Egg shell elements’ concentrations are presented in Table 3. Addition of 200 mg quercetin per kg of diet significantly decreased the concentration, compared to control, of As (14%) and increased the concentration of Fe (9.5%) and Ni (7.5%). Quercetin added to the diet at 400 mg/kg decreased, compared to control, the concentrations of six elements namely As (40%), Cd (22%), Cr (7%), Cu (30%), Se (42%) and V (7%) while increased the levels of Ca (7.5%), Fe (12%), Mg (14%), Ni (13%) and Sb (2-fold). Accordingly, addition of 800 mg quercetin per kg of diet, significantly decreased the concentrations, compared to control, of six elements namely As (56%), Cd (46%), Cr (10%), Cu (25%), Se (43%) and V (14%) while significantly increased the levels of Fe (8%), Ni (11%) and Sb (2.8-fold) (Table 3).

### 3.2. Principal Components Analysis

**Figure 1:**
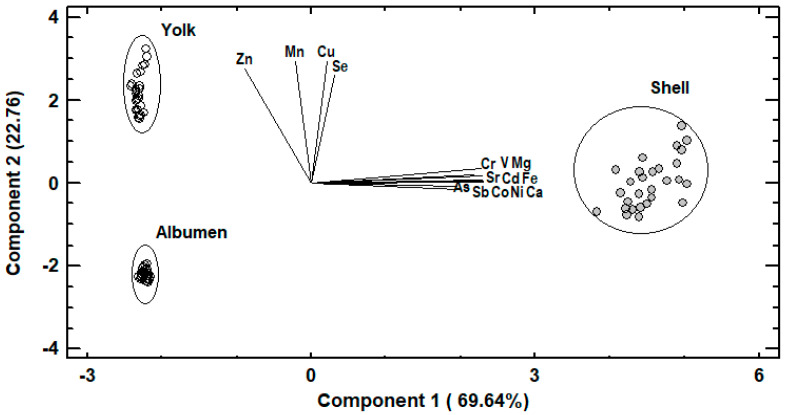
Principal components analysis. Loadings of trace and macro elements for the two first principal components and score plot of albumen, yolk or shell samples.

The concentrations of the 15 selected trace and macro elements in egg albumen, yolk and shell were subjected to principal components analysis (PCA) in order to investigate the relationships between the elements and detect those elements capable of distinguishing samples (Figure 1). PCA resulted in two principal components, which accounted for 92.40% of the total variability. Trace and macro elements in albumen, yolk and shell samples are presented in Figure 1, as a function of both first and second principal components. The first principal component (PC1) explained 69.64% of the total variability and was defined by the elements As, Ca, Cd, Co, Cr, Fe, Mg, Ni, Sb, Sr and V. The aforementioned elements were located away from the axis origin, suggesting that they were well represented by PC1 and closed together indicating a strong positive correlation. Egg shell samples were clustered near them and therefore, they had higher contents of these elements compared to the samples from egg albumen and yolk, which were clustered on the negative side of PC1. The second principal component (PC2) explained another 22.76% of the total variability and was defined by Cu, Mn, Se and Zn, which were placed closed together on the positive side of PC2, indicating a positive correlation. Samples collected from egg yolk were clustered near them, indicating higher contents of these elements compared to the samples from egg albumen and shell. Thus, a very clear separation of albumen, yolk and shell samples was observed.

### 3.3. Discriminant Analysis

**Figure 2:**
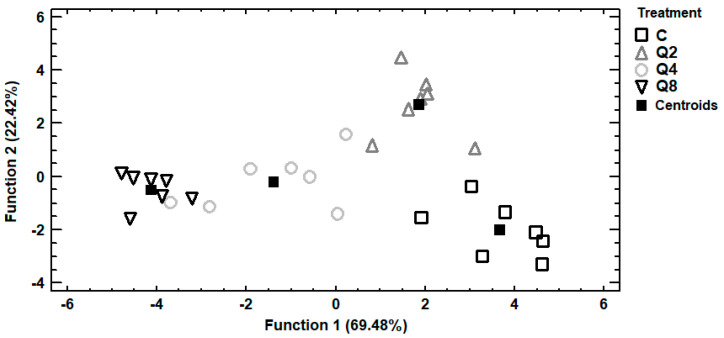
Discriminant plot separating egg albumen samples, according to the dietary treatment, based on trace and macro elements.

**Figure 3:**
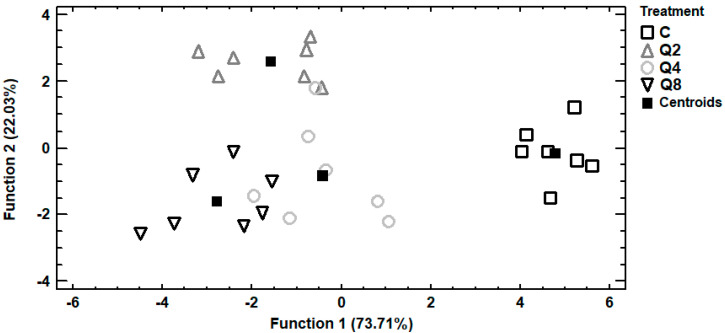
Discriminant plot separating egg yolk samples, according to the dietary treatment, based on trace and macro elements.

**Figure 4:**
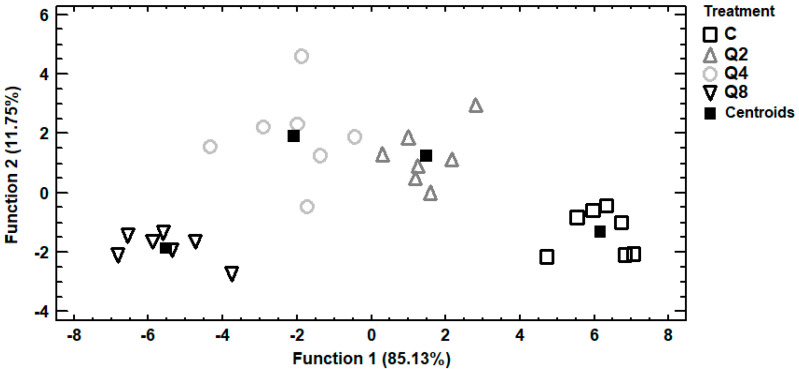
Discriminant plot separating egg shell samples, according to the dietary treatment, based on trace and macro elements.

Trace and macro elements data of egg albumen were subjected to discriminant analysis in order to investigate samples distinguishment according to the four dietary treatments and establishment of those elements capable to distinguish the samples. In Figure 2, it can be seen a discriminant plot of the first two discriminant functions, even though one discriminant function was statistically significant (*p* < 0.001). All the samples were successfully clustered by the dietary treatment and a 100% correct classification was observed. Control samples were placed in the bottom right-hand corner of the plot, far away from all the treated samples. On the contrary, samples from treatment Q8 were clustered on the left side of the plot, having the longest distance from control samples and indicating that the most differentiations in the selected trace and macro elements were observed among these dietary treatments. Samples from treatments Q2 and Q4 were clustered in the middle of the plot and therefore samples from these treatments had intermediate concentrations of the selected elements. In addition, a stepwise discriminant analysis revealed that As, Cd and Cr were mainly responsible for the observed discrimination. Another discriminant plot can be seen in Figure 3, categorizing samples according to the dietary treatment, based on selected trace and macro elements of egg yolk. Samples were successfully clustered by the dietary treatment, although some overlapping between samples from treatments Q4 and Q8 was observed. One discriminant function was statistically significant (*p* < 0.001) for distinguishing the samples and the elements Sb, Se and V contributed the most for this discrimination. The percentage of the samples that were classified into the correct group according to the dietary treatment was 92.9%. Further discrimination, based on selected trace and macro elements of egg shell, was attempted (Figure 4). In this case, control samples were also placed far away from samples of treatment Q8, as in albumen’s discriminant plot and samples from treatments Q2 and Q4 were also clustered in the middle of the plot. One discriminant function was statistically significant (*p* < 0.001) and a 96.4% correct classification was succeeded. Furthermore, a stepwise discriminant analysis revealed that As, Fe, Sb, Se and V were mainly responsible for distinguishing the observations.

## 4. Discussion

### 4.1. Flavonoids in Poultry Nutrition

Quercetin has been shown to be a great in vitro antioxidant [22] that exerts anti-inflammatory effects in rodents [23] and humans [24] and antitumor effects in rodents [25]. Various studies in poultry have indicated that quercetin and its glycosides are metabolized and absorbed in a similar way to mammals [26]. Utilization of quercetin by laying hens enhances their antioxidant status [27,28], reduces yolk [3] and serum cholesterol [27] levels, and modifies the intestinal environment as it decreases cecal microflora populations of total aerobes and coliforms while increases the populations of *Bifidobacteria* [29].

Our earlier results, in agreement with other studies [3], showed that supplementation of hens’ diet with various levels of quercetin for four weeks promoted oxidative stability in a dose dependent manner and was beneficial in improving egg quality [19]. Naringin and hesperidin can be used to alter the elemental profile of the egg by increasing or decreasing the concentration of certain elements in the egg compared to the concentration of those elements in eggs of hens fed a control diet or a control diet supplemented with extra vitamin E [30].

In the present study, the highest contents for the majority of elements were measured in the egg shell compared to yolk and albumen. Moreover, yolk had higher levels than albumen indicating that basic elements are stored in the yolk, where most egg minerals are deposited, and from where they can be effectively transferred via the yolk sac to the developing embryo. Elements accumulated in the albumen may also be accessible during embryogenesis [31,32].

The bioavailability of Zn, Fe, Ca, Mg and *P* from the eggshell was examined in an in vitro study studying the role of citrus flavonoids and reported enhanced bioavailability of these elements by citrus flavonoids [33]. A balance between absorption and excretion of elements exists, mineral homeostasis, and the key role in this balance is assigned to specialized proteins such as Cu- and Zn transporters and Mg channels to maintain [34]. Metallothioneins, that help mammals against heavy metals toxicity and contribute to the homeostasis of certain essential metals, are cysteine-rich protein of low molecular weight, localized to the membrane of the Golgi apparatus that exhibit high binding capacity for both physiological (such as Cu, Se, Zn) and xenobiotic (such as Ag, As, Cd, Hg,) essential and toxic elements [35,36]. Previous studies have indicated a protective effect of quercetin against oxidative damage of erythrocyte membrane which was attributed to its iron chelating activity [37].

### 4.2. Modulation of Egg Metallome

In the present study, supplementation of layers diet with quercetin mostly affected the trace- and macro elements of the egg shell compared to control fed layers. Quercetin supplementation increased the concentrations of five elements (Ca, Fe, Mg, Ni and Sb) while decreased the levels of six elements namely As, Cd, Cr, Cu, Se and V. Solely the concentration of Sb was uniformly increased with quercetin in albumen, yolk and shell compared to control fed layers. Quercetin seemed to affect the homeostasis of trace elements in hen eggs as reflected by changes of egg’s elemental profile in the three egg compartments.

**Scheme 1:**
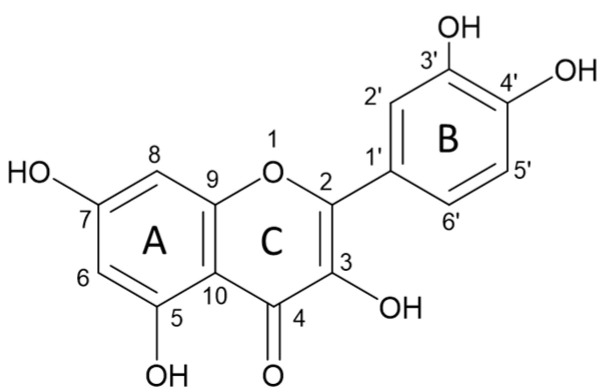
Structure of quercetin.

The creation of complexes of flavonoids with elements may be related to certain effects of flavonoids on trace element homeostasis. Quercetin and other flavonoids have received considerable attention as dietary constituents in the recent years [38]. Numerous studies have indicated that flavonoids possess a wide range of biological activities, such as antiviral, antioxidant, anticancer, antibacterial, and anti-inflammatory. Flavonoids are best known as radical scavengers. Beneficial effects are related to the capacity of accepting free radicals and to exhibiting complexation properties with metal ions [39]. Several studies including but not limited to experimental and theoretical ones have reported that the flavonoid complexation is related to the hydroxyl group on carbon 3 or 5 and the adjacent 4-carbonylgroup in the C ring (Scheme 1) [40].

The results of the current study work are in line with earlier results on rodents that reported a relation between tissue availability of several elements and absorption and membrane transport of some metal ions which can be affected by the formation of chelates and complexes with organic ligands [41]. The present study revealed that deposition of certain elements was higher in eggs from quercetin fed layers compared to those from the control treatment indicating the chelating capacity of quercetin. Previous studies on humans, animal models or plants have shown the formation of such complexes. Markedly, in a study with postmenopausal women, supplementation with Ca in combination with a flavonoid improved bone Ca retention by 5.5% [42]. However, this was not evident in the case of hens in the present study. In addition, in obese rats fed high-fat or high-carbohydrate diets it was noticed that flavonoid supplementation exerted a vasodilatation effect probably by activating large conductance Ca^2+^-activated K^+^ currents in a concentration-dependent manner [43].

Antioxidant but also chelating activities of flavonoids in in vivo biological systems are not straightforward and depend on a variety of factors including: (i) the active concentrations in the target tissue compartment, which are very low, (ii) the absorption efficiency, which is relatively low, (iii) metabolite formation after absorption which could reduce their activities and (iv) the surrounding flavonoid environment which can strongly determine in which extend polyphenols exert their activities [44]. Clearly, further research is required to elucidate the metal complexation properties of different dietary quercetin and/or quercetin metabolites levels in egg.

In the present study, the presence of quercetin resulted in higher Fe concentration in the shell. Flavonoids have been shown to protect against induced oxidation and having strong antioxidant activity and Fe chelating properties. In glutathione depleted mouse erythrocytes, the role of quercetin as an antioxidant against induced oxidation was examined and it was showed markable protection against lipid peroxidation by Fe chelation and erythrocytes penetration [32]. Similarly, Deng et al. [45] examined five flavonoids namely quercetin, hesperidin, naringin, baicalin, and rutin and indicated that their antioxidant function originates from chelating Fe ions and from scavenging peroxyl radicals. Differences between flavonoids may be due to their structure and the site of chelation. It has been reported [46] that the majority of flavonoids display a yield of redox reactions greater with Cu than with Fe, as a result of the lower redox potential of Cu^2+^/Cu^+^ compared with that of Fe^3+^/Fe^2+^. Furthermore, it has been shown that quercetin protected cells against oxidative damages caused by Fe overloading. The anti-carcinogenic effect of quercetin has been linked with the chelating effect of Fe as well as its antioxidant effect. It has been reported that quercetin-metal complexes might possess even greater anticancer and antibacterial activities than free quercetin [9,16]. Nevertheless, our results cannot explain the relationship among quercetin, Fe and potential antioxidant effects since there was no positive correlation between quercetin administration levels and Fe concentrations in egg yolk and egg albumen.

Addition of quercetin, in this study, manifested in altered concentrations of Mg and Cd in specific egg fractions. Earlier studies with Mg and flavonoids indicated formation of quercetin–magnesium complexes. More specifically, the interaction of MgSO_4_.7H_2_O with quercetin was tested and it was reported that the Mg ions significantly modify the chemical properties of quercetin [47]. Though, regarding egg yolk and albumen, Mg levels were not changed by quercetin supplementation. Studies conducted in plants (*Avicennia marina* roots) reported increased absorption rate of Cd under flavonoid amendment [48]. In this aspect, regarding egg albumen, addition of quercetin in the highest dose (Q8) increased Cd levels, opposite to Cd levels in egg shell that were decreased and to egg yolk that remained unaffected. Thus, obtaining clear conclusions regarding mitigation or increase of toxicity is difficult. Nevertheless, further research is required to detect a direct relationship between dietary quercetin levels and metal complexing properties in this flavonoid.

In the present study, the presence of 400 and 800 mg quercetin per kg of diet resulted in lower Cr, Cu and Se concentrations in egg albumen and shell. It has been reported that some flavonoids can also decrease the absorption of trace elements, when included in a diet. For example, addition of grape seed extracts impeded the absorption of Zn and Fe in a dose-dependent manner in intestinal cells [44,49]. Different factors including but not limited to the type of compounds, the dosage and the combination with other compounds affect assimilation and absorption of trace metals by polyphenol compounds [50].

Development of eggs fortified in certain trace elements has been proposed through feed supplementation [30]. Notwithstanding, bioavailability restrictions have led to the usage of appropriate forms of supplements. These limitations could be transcended through chelation with flavonoids rich in phenolic groups. Homeostasis of macro- and trace element has significant features that can be studied by elemental metabolomics through determination of the complete elemental profile [51].

Earlier studies [26] revealed that quercetin and its glycosides can be absorbed and metabolized by chicken. In avian species, similar to mammals, quercetin following absorption can be subjected to glucuronidation, sulfation, and methylation. However, it was reported that incorporation of quercetin or its glycoside in the diet did not increase the antioxidant capacity in plasma or tissues, suggesting that the absorption of quercetin was rather weak or the concentrations of quercetin metabolites in plasma or tissues were not sufficient to significantly increase the antioxidant capacity assayed by the ferric reducing ability of plasma (FRAP) [26].

The present study revealed that complex interactions exist that cannot be solely attributed to chelation of elements. Feeding trials at different levels of flavonoids may further illuminate interactions. Such kind of future studies with fine tuning of the elemental supplementation and simultaneous adjustment of flavonoid levels may lead to creation of tailor-made eggs, to address specific needs like Fe enriched diets [52]. Although data presented here derived from a flavonoid used in its pure chemical form, various agricultural by-products of the olive oil-, fruit juice- and wine industry are rich in phenolics and flavonoids could be also used.

The egg shell metallome alteration due to quercetin dietary supplementation found in the present study may be considered favorable from the avian embryonic development perspective. Macro- and trace elements, essential for embryonic growth such as Ca, Fe and Mg, were increased in the eggshells of quercetin fed hens in comparison to control fed ones. At the same time, elements that are considered toxic at high levels such as Cd, Cr, and Cu were decreased. The eggshell is a macro- and trace nutrient pool for the developing avian embryo and therefore its composition affects embryo development. Further research is required to elucidate how eggshell metallome may affect embryonic and post-hatch development.

## 5. Conclusions

In conclusion, quercetin supplemented to the diet of layers can affect the elemental profile of egg because a differentiation in the deposition of certain elements in the egg constituents, compared to that of hens fed a control diet, was noted. Quercetin added at 200, 400 and 800 mg per kg of hen feed increased the concentration of Sb (2-140-fold) while reduced that of Cr (7–15%) and Se (34–43%) in both egg shell and albumen. Moreover, it increased As (18–24%), Cd (32–34%) in albumen and V (7.5–9%) in yolk, while compared to the control, reduced As (14–56%), Cd (22–46%), Cr (7–10%), Cu (25–30%) and V (7–14%) and also raised Ca (7.5%), Fe (8–9.5%), Mg (14%) and Ni (7.5–13%) in egg shell.

## Figures and Tables

**Table 1:** The effect of dietary supplementation with quercetin on selected trace- and macro-elements concentrations in laying hens’ egg albumen (*n* = 7).

			Treatment			
Elements (ppb)	C	Q2	Q4	Q8	SEM	*p*-Value
As	4.27 ^a^	4.40 ^a^	5.06 ^b^	5.31 ^b^	0.113	<0.001
Ca	7.15 × 10^4^	6.78 × 10^4^	5.59 × 10^4^	5.19 × 10^4^	7.41 × 10^3^	0.214
Cd	2.77 ^a^	3.73 ^b^	3.14 ^ab^	3.67 ^b^	0.233	0.022
Co	1.31	1.60	1.33	1.33	0.099	0.147
Cr	89.5 ^c^	87.2 ^c^	81.2 ^b^	76.3 ^a^	1.386	<0.001
Cu	276.7	244.3	246.7	248.7	15.81	0.446
Fe	5.21 × 10^3^	7.31 × 10^3^	6.65 × 10^3^	5.50 × 10^3^	806.6	0.246
Mg	1.06 × 10^5^	0.908 × 10^5^	1.02 × 10^5^	0.959 × 10^5^	4.74 × 10^3^	0.154
Mn	58.5	65.4	61.7	62.7	9.014	0.959
Ni	40.7	37.0	37.9	40.8	2.207	0.519
Sb	0.019 ^a^	0.014 ^a^	0.585 ^a^	2.69 ^b^	0.426	0.001
Se	88.6^c^	84.2 ^bc^	67.7 ^ab^	58.0 ^a^	6.672	0.011
Sr	150.3	152.9	132.4	146.4	9.593	0.454
V	78.8	81.6	82.9	84.3	1.631	0.129
Zn	1.68 × 10^3^	1.41 × 10^3^	1.18 × 10^3^	1.49 × 10^3^	143.8	0.129

C: no additive, Q2, Q4 and Q8: 200, 400 and 800 mg quercetin per kg of feed, respectively. ^a,b,c^ means in a row sharing no common superscript differ significantly (*p* < 0.05).

**Table 2:** The effect of dietary supplementation with quercetin on selected trace- and macro-elements concentrations in laying hens’ egg yolk (*n* = 7).

			Treatment			
Elements (ppb)	C	Q2	Q4	Q8	SEM	*p*-value
As	3.73	3.61	3.69	3.71	0.110	0.887
Ca	1.03 × 10^6^	1.11 × 10^6^	1.13 × 10^6^	1.11 × 10^6^	3.73 × 10^4^	0.291
Cd	3.93	5.47	4.61	4.31	0.437	0.108
Co	3.30	3.44	3.73	3.41	0.134	0.164
Cr	148.1	155.7	152.6	150.7	4.072	0.614
Cu	1436	1473	1578	1509	43.46	0.153
Fe	6.89 × 10^4^	7.20 × 10^4^	7.06 × 10^4^	6.55 × 10^4^	3.86 × 10^3^	0.672
Mg	1.25 × 10^5^	1.35 × 10^5^	1.29 × 10^5^	1.33 × 10^5^	3.77 × 10^3^	0.307
Mn	1141	1169	1221	1179	79.01	0.914
Ni	66.7	70.1	67.9	68.0	2.220	0.754
Sb	0.077 ^a^	0.408 ^ab^	0.582 ^b^	0.613 ^b^	0.116	0.012
Se	232.9	316.4	236.8	301.5	29.76	0.125
Sr	696.9	752.9	725.0	697.4	34.28	0.618
V	104.7 ^a^	114.2 ^b^	114.4 ^b^	112.7 ^b^	2.173	0.016
Zn	4.00 × 10^4^	4.15 × 10^4^	4.22 × 10^4^	4.20 × 10^4^	1.21 × 10^3^	0.577

C: no additive, Q2, Q4 and Q8: 200, 400 and 800 mg quercetin per kg of feed, respectively. ^a,b^ means in a row sharing no common superscript differ significantly (*p* < 0.05).

**Table 3:** The effect of dietary supplementation with quercetin on selected trace- and macro-elements concentrations in laying hens’ egg shell (*n* = 7).

			Treatment			
Elements (ppb)	C	Q2	Q4	Q8	SEM	*p*-value
As	16.63 ^d^	14.21 ^c^	9.84 ^b^	7.24 ^a^	0.857	<0.001
Ca	2.27 × 10^8 a^	2.39 × 10^8 ab^	2.44 × 10^8 b^	2.39 × 10^8 ab^	4.00 × 10^6^	0.040
Cd	70.2 ^c^	63.8 ^bc^	54.5 ^b^	37.7 ^a^	4.098	<0.001
Co	270.6	281.7	288.3	270.6	5.699	0.097
Cr	692.9 ^b^	663.1 ^ab^	642.9 ^a^	625.2 ^a^	14.99	0.024
Cu	1218 ^b^	996.0 ^ab^	843.3 ^a^	905.1 ^a^	80.16	0.016
Fe	1.79 × 10^6 a^	1.96 × 10^6 b^	2.00 × 10^6 b^	1.93 × 10^6 b^	4.20 × 10^4^	0.033
Mg	2.87 × 10^6 a^	2.99 × 10^6 a^	3.28 × 10^6 b^	3.06 × 10^6 a^	8.66 × 10^4^	0.020
Mn	491.1	632.1	514.6	488.6	48.70	0.148
Ni	9.49 × 10^3 a^	1.02 × 10^4 b^	1.07 × 10^4 b^	1.06 × 10^4 b^	192.3	<0.001
Sb	33.86 ^a^	47.70 ^a^	72.26 ^b^	94.88 ^c^	6.907	<0.001
Se	273.9 ^b^	221.9 ^ab^	156.8 ^a^	154.2 ^a^	27.78	0.015
Sr	1.05 × 10^5^	1.11 × 10^5^	1.10 × 10^5^	1.07 × 10^5^	3.42 × 10^3^	0.506
V	663.9 ^c^	654.7 ^c^	618.4 ^b^	572.2 ^a^	9.019	<0.001
Zn	6.95 × 10^3^	9.02 × 10^3^	6.51 × 10^3^	6.11 × 10^3^	888.4	0.124

C: no additive, Q2, Q4 and Q8: 200, 400 and 800 mg quercetin per kg of feed, respectively. ^a,b,c^ means in a row sharing no common superscript differ significantly (*p* < 0.05).

## Data Availability

Data is contained within the article.